# Microfluidic Synthesis of Indomethacin-Loaded PLGA Microparticles Optimized by Machine Learning

**DOI:** 10.3389/fmolb.2021.677547

**Published:** 2021-09-22

**Authors:** Safa A. Damiati, Samar Damiati

**Affiliations:** ^1^ Department of Pharmaceutics, Faculty of Pharmacy, King Abdulaziz University, Jeddah, Saudi Arabia; ^2^ Department of Biochemistry, Faculty of Science, King Abdulaziz University, Jeddah, Saudi Arabia; ^3^ Division of Nanobiotechnology, Department of Protein Science, Science for Life Laboratory, School of Engineering Sciences in Chemistry, Biotechnology and Health, KTH Royal Institute of Technology, Stockholm, Sweden

**Keywords:** microfluidics, machine learning, polymeric particles, PLGA, pharmaceutics

## Abstract

Several attempts have been made to encapsulate indomethacin (IND), to control its sustained release and reduce its side effects. To develop a successful formulation, drug release from a polymeric matrix and subsequent biodegradation need to be achieved. In this study, we focus on combining microfluidic and artificial intelligence (AI) technologies, alongside using biomaterials, to generate drug-loaded polymeric microparticles (MPs). Our strategy is based on using Poly (D,L-lactide-co-glycolide) (PLGA) as a biodegradable polymer for the generation of a controlled drug delivery vehicle, with IND as an example of a poorly soluble drug, a 3D flow focusing microfluidic chip as a simple device synthesis particle, and machine learning using artificial neural networks (ANNs) as an in silico tool to generate and predict size-tunable PLGA MPs. The influence of different polymer concentrations and the flow rates of dispersed and continuous phases on PLGA droplet size prediction in a microfluidic platform were assessed. Subsequently, the developed ANN model was utilized as a quick guide to generate PLGA MPs at a desired size. After conditions optimization, IND-loaded PLGA MPs were produced, and showed larger droplet sizes than blank MPs. Further, the proposed microfluidic system is capable of producing monodisperse particles with a well-controllable shape and size. IND-loaded-PLGA MPs exhibited acceptable drug loading and encapsulation efficiency (7.79 and 62.35%, respectively) and showed sustained release, reaching approximately 80% within 9 days. Hence, combining modern technologies of machine learning and microfluidics with biomaterials can be applied to many pharmaceutical applications, as a quick, low cost, and reproducible strategy.

## Introduction

Indomethacin (IND) is a non-steroidal anti-inflammatory drug (NSAID), used as an analgesic and anti-pyretic. IND is a poorly water soluble drug (∼3 μg/ml), is light sensitive, crystalline, poorly water soluble, and has a moderate half-life of 4–5 h (Song et al., 2002; Ziltener et al., 2010; NCBI 2021). Besides its ability to relieve inflammation and pain, clinical evidence has reported on the role of IND alone or combined with chemotherapy in preventing cancers, such as stomach, colorectal and prostate cancer (Hull et al., 2003; Chiou et al., 2014; Liu et al., 2019). However, the limited utilization of IND is attributed to its ability to cause gastrointestinal ulceration and haemorrhage (Kang et al., 2008). Hence, attempts have been made to generate controlled release carriers of IND to reduce its adverse effects, improve its solubility, and increase its bioavailability. Encapsulation of IND within a polymeric matrix would allow its slow release in a controlled manner into the gastrointestinal tract, and decrease the doses required because of the sustained release of the loaded drug (Vilos and Velasquez 2012; Shams et al., 2017).

Drug delivery vehicles based on biodegradable and biocompatible polymers are widely used, and several examples have already been approved by the FDA, such as leuprolide (Lupron Depot) and triptorelin (Trelstar) (Xing et al., 2019; Sung and Kim 2020). Poly (D, L-lactide-co-glycolide) (PLGA) is the most versatile polymer used as a matrix material for many biopharmaceutical applications. PLGA particles can be loaded with proteins, siRNA, and many drugs (Xie and Smith 2010). Furthermore, these particles are effective via various delivery routes, including intramuscular injection, inhalation, and oral (Xie and Smith 2010; Xing et al., 2019). Particle size and particle size distribution are significant factors influencing particle performance. Particles of a small size and narrow size distribution offer high stability and improve the shelf-life of the final particles. Many techniques are already in use to synthesize drug-loaded particles, but some traditional methods suffer from various limitations, such as production of polydisperse polymeric particles. Microfluidics has offered an effective alternative to traditional methods to fabricate polymeric microparticles. Nowadays, microfluidics is an effective tool to generate microparticles with high monodispersity, precisely tunable structures, and excellent encapsulation efficiency (Damiati et al., 2018; Dashtimoghadam et al., 2020). Microfluidic chips are either lab-made or commercial products made of glass, polymers, or polydimethylsiloxane (PDMS), and consist of rectangular microchannels in different dimensions that are constructed by lithography (Damiati et al., 2018; Dewandre et al., 2020). Still, microfluidics is not without its own limitations, such as the need for extensive laboratory optimization. Hence, combining recent technologies of microfluidics and artificial intelligence (AI) offers a promising method of fabrication of monodisperse particles with well-controlled properties. Machine learning is an AI technique whereby computers can adjust their actions (e.g., making predictions). In addition, artificial neural networks (ANNs) are commonly used machine learning techniques used in pharmaceutics because of their powerful ability to model nonlinear relationships (Damiati et al., 2017; Mendyk et al., 2020). A typical ANN structure consists of input, hidden, and output layers. Through iterative representation of examples, ANN learning occurs (Damiati 2020). ANN has been utilized for predication of particle sizes generated by microfluidics (Damiati et al., 2020).

Here, we present microfluidic generation of IND-loaded PLGA droplets based on a flow focusing technology in a rapid manner. We developed an in silico model using ANNs to predict the size of blank PLGA droplets generated by a 3D flow focusing microfluidic chip. After conditions optimization, the obtained results were employed to generate IND-loaded PLGA droplets. The chosen system was based on generation of droplets of an acceptable small size, sufficient quantity, and narrow size distribution. Finally, generated IND-loaded PLGA microparticles resulting from the droplets drying were characterized for their physicochemical characteristics, drug encapsulation efficiency, drug loading, and drug release profiles.

## Experimental Section

### Materials

A hydrophilic 3D flow focusing microfluidic glass chip with 100 μm channels (3,200,433) was purchased from Dolomite Microfluidics (United Kingdom) and used to create blank or IND-loaded PLGA droplets. Indomethacin, PLGA (lactide:glycolide 50:50), polyvinyl acetate (PVA) (MW 9,000–10,000, 80% hydrolyzed), and Dichloromethane (DCM) were supplied by Sigma Aldrich (United Kingdom). To monitor droplets generation, a digital microscope (Dolomite, United Kingdom) was used. Fluids were injected and controlled into the microfluidic device by a flow control system (Fluigent, France).

### PLGA Droplet/Microparticle Preparation

Blank and IND-loaded PLGA MPs were manufactured with a 3D flow focusing microfluidic device. Initially, to generate blank PLGA droplets, different PLGA solutions were prepared at different concentrations (1, 2, 5% w/w). For each solution, an appropriate amount of PLGA was dissolved in DCM with continuous mechanical stirring to completely dissolve the polymer. To prepare droplets, 1% w/v PVA was used as an aqueous continuous phase and injected separately into two microfluidic inlets, whereas PLGA in DCM was the dispersed phase and injected into the central inlet. To evaluate the impact of different microfluidic production parameters, continuous and dispersed phases were injected at different flow rates. After optimizing the droplet generation conditions, IND-loaded PLGA droplets were prepared similarly to the blank ones, except the dispersed phase was DCM containing 2% PLGA and 0.5% IND. The generated droplets were collected in PVA aqueous solution to prevent droplet coalescence. Evaporation of DCM and generation of MPs were done using a rotary evaporator under reduced pressure at RT. Solidified blank or IND-loaded PLGA MPs were collected by centrifugation at 1,500 rpm for 5 min, and rinsed with deionized water to remove excess PVA.

### In Silico Prediction of PLGA Droplet Size

The multilayer perceptron (MLP) ANN was employed using Statistica, version 13.3 software (TIBCO Sofware Inc, 2017). The ANN model was trained using the experimental data for the generation of blank PLGA droplets. A neural network consisting of an input, hidden, and output layer, with 3-6-1 neurons, respectively, was found to be adequate for predicting PLGA droplet size. The parameters chosen as inputs for the ANN were simple, including PLGA concentration and the flow rates of both PLGA and aqueous phases. The output layer consisted of the measured PLGA droplet sizes. All input features are continuous and were normalized to the range 0–1. Learning parameters were varied to optimize training performance and prediction accuracy. Tanh and Identity were used as the hidden and the output activation functions, respectively. The total dataset consisted of 23 cases, which has been further divided into ∼60, 20, and 20% for the training, test, and validation sets (Table 1).

**TABLE 1:** ANN training, test, and validation datasets.

Case no	PLGA conc. (%)	Flow rate PLGA (µL/min)	Flow rate PVA aq. Phase (µL/min)	Droplet size (µm) ± SD^a^	Dataset
1	1	3.70	9.20	72.21 ± 0.8	Training
2	1	4.90	9.20	77.68 ± 0.8	Validation
3	1	6.10	9.20	81.79 ± 1.3	Training
4	1	7.40	9.20	92.21 ± 1.1	Test
5	1	8.60	9.20	97.44 ± 1.5	Training
6	1	11.01	9.20	101.67 ± 0.9	Training
7	1	12.40	9.20	103.87 ± 0.8	Validation
8	1	13.80	9.20	108.76 ± 1.8	Training
9	1	4.90	18.40	13.18 ± 0.5	Test
10	1	11.01	18.40	78.42 ± 0.9	Training
11	1	14.70	18.40	99.29 ± 0.9	Training
12	2	2.50	9.20	37.63 ± 0.6	Training
13	2	3.70	9.20	38.82 ± 0.5	Validation
14	2	4.90	18.40	12.89 ± 0.4	Training
15	2	7.40	18.40	56.52 ± 0.8	Training
16	2	11.01	18.40	87.11 ± 0.7	Test
17	2	14.70	18.40	93.74 ± 0.6	Training
18	2	19.64	18.40	92.99 ± 1.3	Training
19	5	3.70	18.40	16.35 ± 0.8	Training
20	5	7.40	18.40	50.15 ± 0.8	Training
21	5	11.01	18.40	73.59 ± 0.7	Test
22	5	14.70	18.40	81.79 ± 1.4	Validation
23	5	19.64	18.40	97.14 ± 1.3	Train training

a Droplet size averages were measured by using ImageJ. For each sample, the mean size was calculated based on the measurements of 15 randomly chosen droplets.

### Characterization

Droplet/particle size distribution and morphology was examined using microscopic image analysis. The average particle size and size distribution were analyzed using ImageJ Software Version1.52a (NIH, US). Distribution is expressed as the polydispersity index (PDI), and was calculated with the following formula:

$$PDI\;\%=\frac{standard\;deviation}{average\;droplet\;diameter}\;\times 100$$

A low PDI value indicates a narrow size distribution (monodisperse particles), while a high PDI value indicates a wide size distribution (polydisperse particles). The size distribution is reflected narrow for a PDI value < 5%.

### Determination of IND Content in the PLGA MPs

The IND loading was determined by dissolving 10 mg IND-loaded PLGA MPs in DMSO, and then diluting with PBS (pH 7.4). The drug content of the MPs was quantified by measuring the UV absorbance at 320 nm, and then the results were compared to a standard curve of known concentrations of IND (Supplementary Figure S1). Drug loading (DL%) and encapsulation efficiency (EE%) were calculated using the following formulas:

$$DL\;\%=\;\frac{\text{mass of drug in MPs}}{total\;mass\;of\;MPs}\;\times 100$$

$$EE\;\%=\;\frac{\text{mass of drug in MPs}}{initial\;mass\;of\;drug}\;\times 100$$

### *In Vitro* Drug Release Study

UV spectroscopy was used to monitor the stability of the produced MPs, in terms of IND release. The release behavior of 10 mg IND-loaded PLGA MPs was determined by dialysis against 10 ml PBS (pH 7.4) at 37°C, with continuous agitation. At predetermined time intervals, 100 μL of PBS was withdrawn and replaced with the same amount of fresh PBS to maintain the dissolution medium at a constant volume. The amount of released IND was determined by UV spectrophotometry.

## Results and Discussion

### Optimization of the Process Parameters for the Generation of Blank PLGA Droplets in the Microfluidic Chip

**FIGURE 1:**
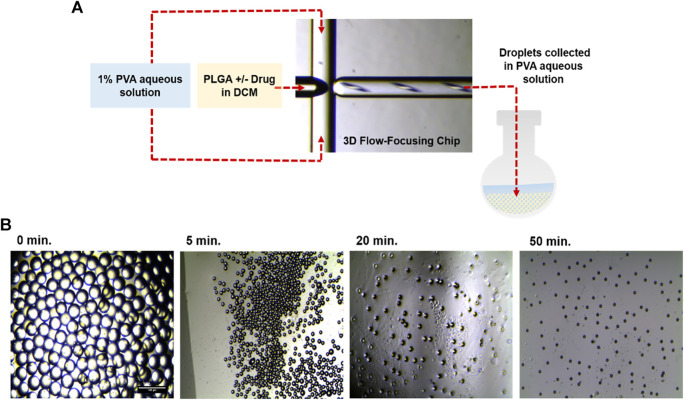
**(A)** Illustration of the experimental procedure of monodisperse PLGA droplet generation at the orifice of the flow-focusing region of the microfluidic chip. **(B)** Optical microscopic images showing, from left to right: (i) PLGA droplets before, (ii,iii) during, (iv) and after DMC evaporation, resulting in generation of PLGA MPs.

Generation of monodisperse particles is a challenging task that arises the impact of particle sizes on the drug release kinetics. Thus, microfluidic devices are attracting more attention due to their ability to control the physical properties of generated particles, including size, dispersity, and shape. In the current study, highly uniform PLGA droplets, either blank or loaded with IND, were synthesized using a 3D flowfocusing microfluidic chip. Figure 1 shows the process of generation and solidification of PLGA droplets with or without IND, monitored by optical microscopy at different time intervals. Initially, prior to drug loading, engineering of blank PLGA droplets was investigated both experimentally and computationally to assess the effect of PLGA concentration and microfluidic flow rates of the continuous and disperse phases on generation of monodisperse droplets with a low PDI value.

#### PLGA Concentration

**FIGURE 2:**
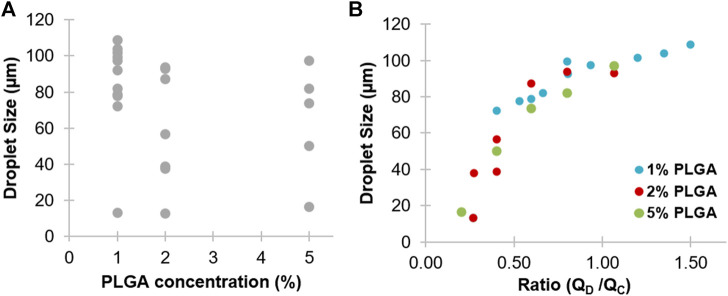
The experimental optimization of PLGA droplet size generated by the 3D flow focusing microfluidic chip. (**A)** Size dependence of droplets at different PLGA concentrations (1, 2, and 5% w/v). (**B)** Size dependence of droplets at different relative flow rates of the disperse phase (Q_D_) for the PLGA solution and continuous phase (Q_C_) for 1% v/v PVA. Droplet size was measured by ImageJ.

The generated PLGA droplets with polymer 50:50 and a 3D flow focusing chip showed a reduction in particle size with increasing PLGA concentration, which may be attributed to changing the viscosity of the solution (Figure 2A). At 1% PLGA, the generated droplet sizes ranged from 13 to 108 µm, depending on input flow rates. Increasing PLGA concentrations to 2–5% led to a size reduction of approximately 13–93 and 16–97 µm, respectively. However, a high concentration of PLGA solution has high viscosity and high interfacial tension, which makes breaking of the flow of the dispersed phase to generate droplets harder (Kim et al., 2013). Further, Roces et al. (2020) showed that generation of nanoparticles using PLGA 50:50 has the smallest particle sizes among other PLGA ratios (75:25 and 85:15).

#### Flow Rates of Disperse and Continuous Phases

Generation of PLGA droplets was tuned by changing the flow rates of the disperse and continuous phases at different PLGA concentrations. The relationship between the size of PLGA droplets and ratio between the two phases is shown in Figure 2B. Above or under the tested ranges, almost no droplets were produced and either single phase or co-laminar flow was observed. Among all generated droplets, the mean of the smallest PLGA droplets was 12.89 ± 0.4 µm, with a standard deviation of 0.4, generated by using 2% PLGA at flow rates ∼5 and ∼20 µL/min for the disperse phase and for the continuous phase, respectively. In contrast, the largest particles (108.76 ± 1.8 µm) were generated using 1% PLGA concentration at flow rates ∼14 and ∼9 µL/min for the disperse phase and for the continuous phase, respectively. However, a high flow rate of the continuous phase led to limitations in the quantity of the generated particles.

### In Silico ANN Model for Prediction of Blank PLGA Droplet Size

**FIGURE 3:**
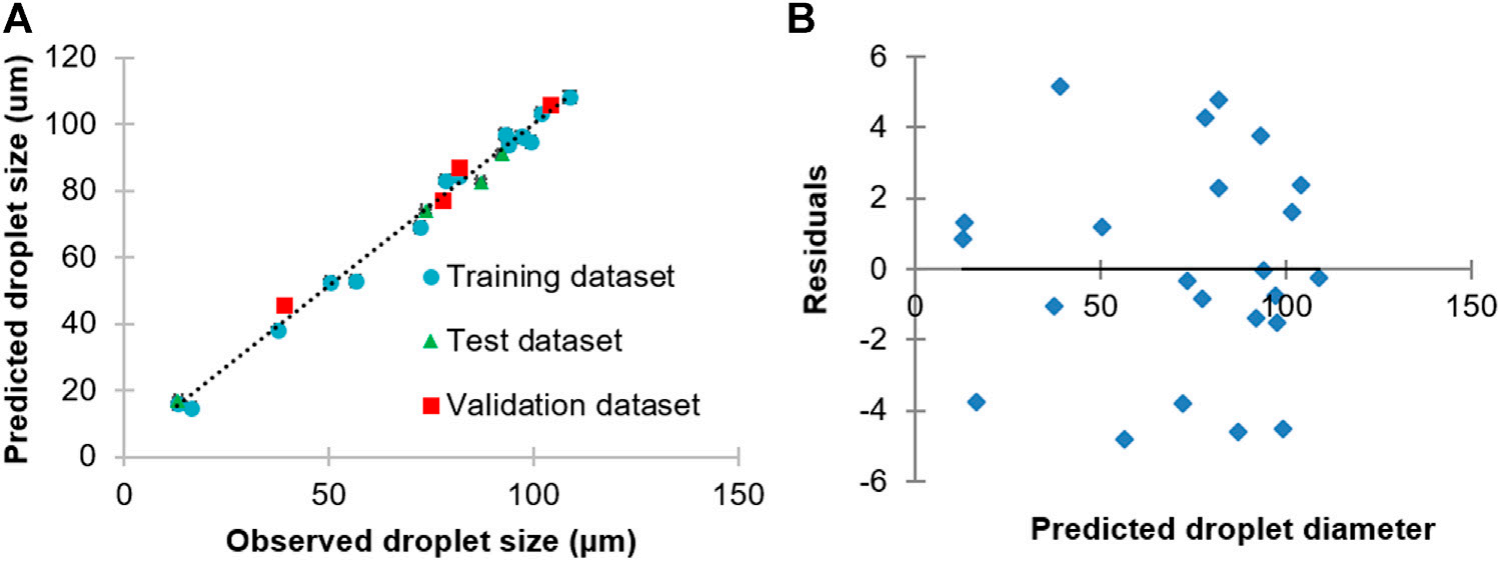
**(A)** Correlation between observed and predicted PLGA droplet diameter generated using a 3D flow focusing droplet chip. **(B)** Plots of residuals for regression of predicted vs. observed PLGA droplet sizes using the ANN model.

Machine learning is a popular AI technique. ANNs, in particular, are commonly used machine learning technique used in pharmaceutical applications because of its powerful ability to model nonlinear relationships (Damiati, 2020). In our study, experimental data from the generation of blank PLGA droplet sizes were used as the target output to train an ANN, with input data furnished by the corresponding PLGA concentration, and PLGA and PVA flow rates (Figure 3A). The developed ANN offered highly accurate predictions of PLGA droplet sizes with residuals randomly scattered in the range ±5 µm (Figure 3B). The correlation of the observed and predicted droplet size data of the whole dataset was high (*r* ^2^ = 0.990). The correlations of the predicted and observed droplet size of the training, test and validation datasets were 0.992, 0.997, and 0.990, respectively. The sensitivity analysis results of the trained ANN model provided key information on the relative importance of the input parameters in defining droplet sizes. By this means, it turned out that the order of importance of the input parameters was: PLGA flow rate > aqueous phase flow rate > PLGA concentration. Hence, the obtained data from the in silico and experimental protocol for PLGA MPs synthesis can be used further to generate drug-loaded particles at a desired size by easy, quick, and economical means.

### Loading of IND as a Model Drug Into PLGA MPs

**FIGURE 4:**
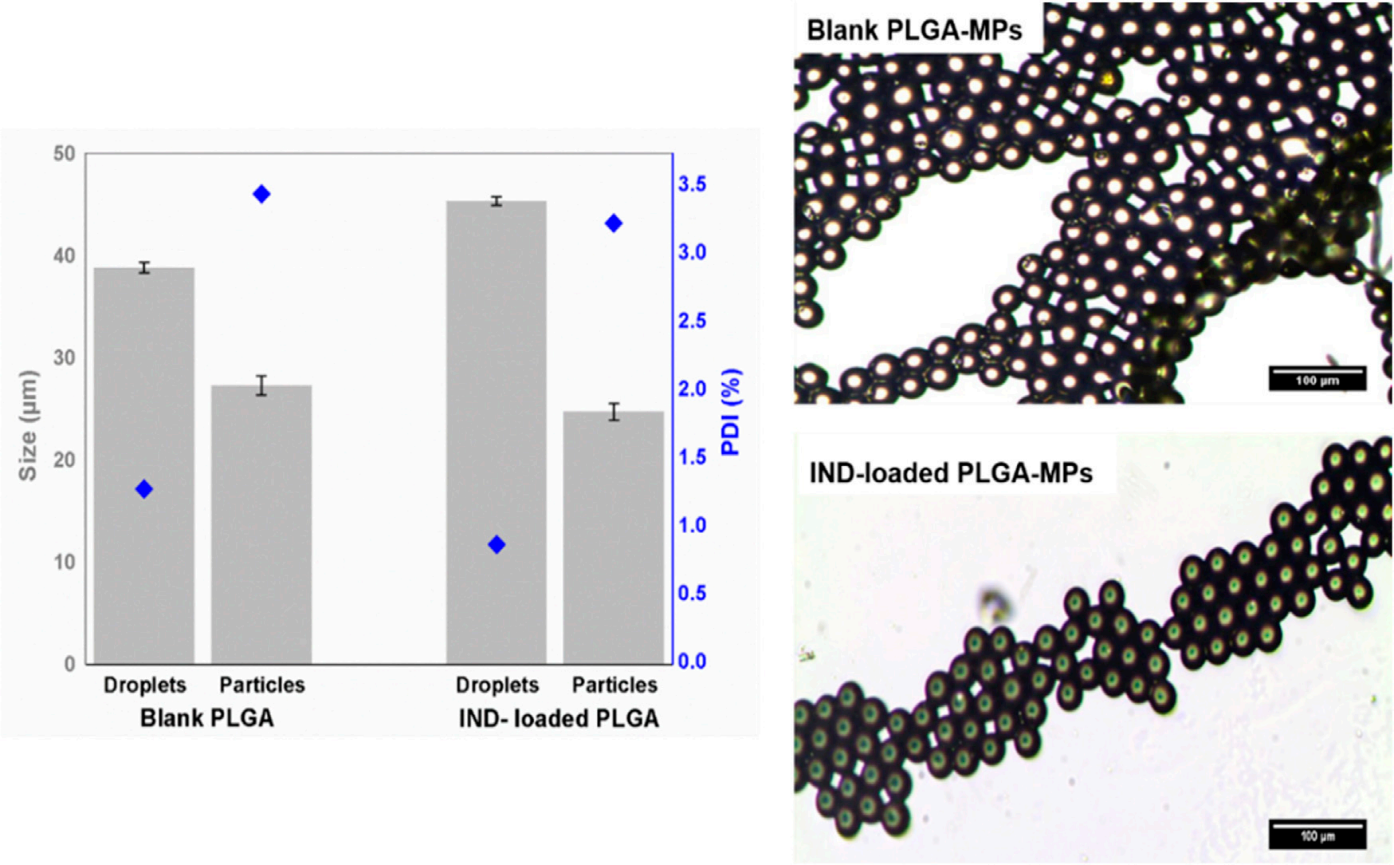
Physicochemical characteristics (size (bars) and PDI (diamonds)) and microscopic images of blank and IND-loaded PLGA MPs generated by the 3D microfluidic chip. The results are represented by the mean ± SD of three independent measurements.

Synthesis of IND-loaded PLGA droplets were fabricated utilizing the same 3D flow-focusing system described above for blank droplets. On the basis of the obtained results from prediction and experimental generation of the blank PLGA droplets, we chose a concentration of 2% (w/v) PLGA and flow rates of 3.7 and 9.2 µL/min for the disperse and continuous phases, respectively, as optimal conditions to generate size-tunable IND-loaded droplets. The chosen conditions were able to generate blank PLGA droplets at the size of 38.82 ± 0.5 µm, and IND-loaded PLGA droplets at size of 45.35 ± 0.4 µm (Figure 4). However, generation of IND-loaded PLGA droplets was not affected by adding IND to the disperse phase, but significantly increased droplet size by around 16.82%. Upon DMC evaporation, the droplets were solidified and shrunk by 30 and 45% for the initial blank and loaded PLGA diameters, respectively. Slow evaporation of the organic solvent from droplets resulted in complete annealing of the polymer which improves particle stability and leads to slower degradation rates (Allison 2008). Converting droplets to particles led to a significant increase in the PDI values, but still all formulations were monodisperse with low PDI values (<5%). A narrow size distribution confirms generation of a homogeneous droplet/particle size population.

### Drug Loading (DL %) and Encapsulation Efficiency (EE %)

DL and EE are two parameters considered crucial parameters to assess the properties of drug-loaded MPs and drug cumulative release profiles. Generated IND-loaded MPs with an average size of 45.35 µm showed an IND loading content of 7.79%, while the EE% was 62.35%. A possible explanation for these results is that IND-loaded PLGA droplets have a relatively small size, which means a high surface:volume ratio. Before solidification and formation of IND-loaded PLGA MPs, loos of the drug occurs at the droplet surface, and leaking to the external aqueous phase leads to a low DL%. Once the polymer droplets solidify, IND is entrapped in the PLGA matrix and this stops drug loss. Similar results were obtained by Chen et al. (2017) for Gefitinib-loaded PLGA microspheres with different size-fractions. In general, high EE reflects the affinity between IND and PLGA, and the hydrophobic nature of IND which increases its encapsulation into polymer particles (Tomoda et al., 2012). However, an improved DL% can be achieved by increasing the initial drug loading, but this may increase particle size, whereas increasing EE% can be achieved by lowering the polymer concentration within the formulation (Roces et al., 2020). Furthermore, when the solubility parameters of the drug and polymer are close, it increases compatibility between them and cause loading of more of the drug. Here, the solubility parameters of PLGA and IND are 28 and 24 MPa, respectively, which enhances drug loading into the PLGA MPs (Liu et al., 2005).

### *In Vitro* IND Release Study

**FIGURE 5:**
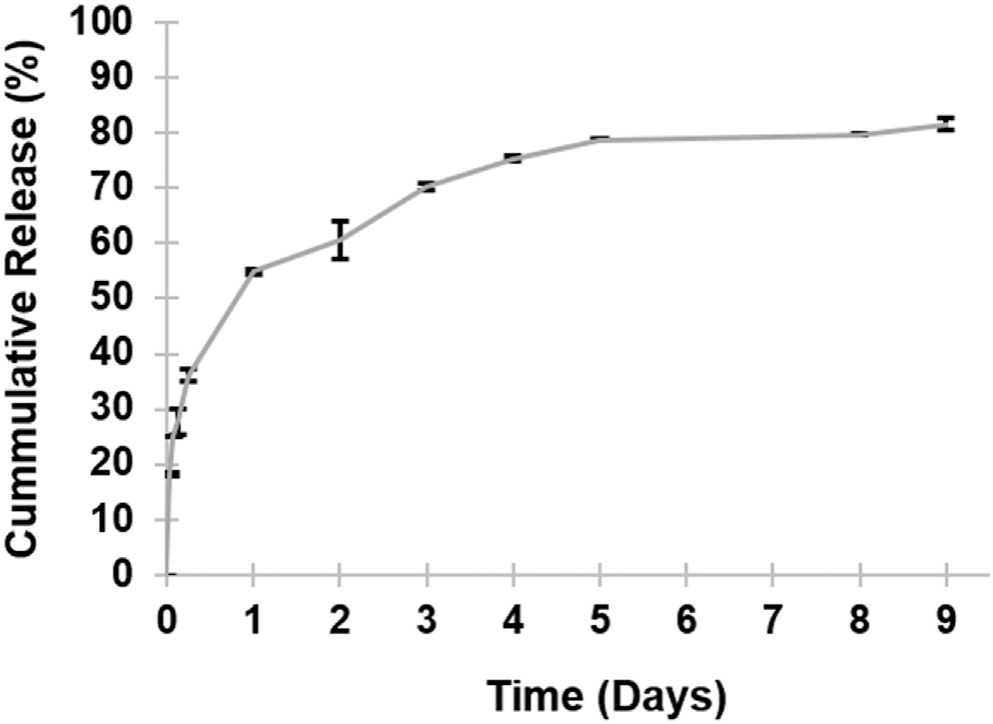
Kinetic release profile of *in vitro* cumulative indomethacin from PLGA MPs over nine days in the experimental environment (PBS pH 7.4, 37°C) using a dialysis system. The released IND was monitored by the UV-Vis spectroscopic method (*n* = 3).

The release behavior of IND from PLGA MPs was evaluated in an experimental environment of PBS at pH 7.4 and at 37°C. The time dependence of the percentage of cumulative drug release from the IND-loaded PLGA MPs is shown in Figure 5. The drug release pattern followed biphasic drug release kinetics. The initial burst phase was up to 36% within 6 h, followed by accumulative release of >80% after 9 days. The delayed release may be attributed to slow diffusion of IND entrapped within the core of PLGA MPs into the dissolution medium.

The composition of a polymer governs the hydrophilicity and rate of degradation of a polymeric carrier system. Increasing the glycolic acid percentage in the oligomers accelerates the weight loss of a polymer. Compared to PLGA 75:25, PLGA 50:50 has a faster degradation that may be attributed to higher hydrophilicity and preferential degradation of the glycolic acid proportion. Moreover, PLGA 50:50 is more amorphous in nature (Makadia and Siegel, 2011; Tsai et al., 2013). All these factors may contribute to faster drug release from PLGA 50:50 particles. However, the loading amount of a drug is significantly influenced by the cumulative release profile. After a large initial burst, a high loading amount leads to a slow and pseudo-linear release. When more drug is distributed near the surface area of the MPs, a higher initial release and faster release rate are achieved. In contrast, a low drug-loading amount follows a gradual increase in release as a function of time (Liu et al., 2006). However, controlling the drug release rate from biodegradable polymers occurs via numerous mechanisms. In the case of the matrix structure, drug release depends on desorption of the surface-adsorbed drug, diffusion of the drug through a polymeric matrix, polymer matrix erosion, and a combination of erosion and diffusion processes (Soppimath et al., 2001).

## Conclusion

This study proposes a successful strategy to generate monodisperse drug-loaded MPs using modern technologies. Microfluidic technology provides manipulation and precise control of PLGA MPs, while machine learning predicts their size, which is a key factor for particle stability. The obtained results showed that microfluidic and AI control parameters, including polymer concentrations and flow rates of dispersed and aqueous phases, can be adopted for generation of well-defined polymeric particles. Our proposed strategy is efficient, rapid, and can be used as a guide to generate polymeric MPs at a desired size, which can be further used to encapsulate drugs with poor aqueous solubility and high toxicity. The produced IND-loaded PLGA MPs exhibited uniformed sizes and morphology, narrow particle size distribution, good encapsulation efficiency, and sustained release behavior. Such a platform could be useful for drug encapsulation into polymeric particles, thus providing a promising drug delivery system for clinical applications. Hence, merging advanced technologies such as microfluidics and machine learning can significantly support developing and modifying new therapeutic agents while reducing the technical difficulties that may act as obstacles in pharmaceutical research. It offers a quick, low cost, and reproducible strategy. However, further *in vivo* investigations are needed in future.

## Data Availability

The original contributions presented in the study are included in the article/Supplementary Material, further inquiries can be directed to the corresponding author.
