# Supplementary figures and images for: Microfluidic Synthesis of Indomethacin-Loaded PLGA Microparticles Optimized by Machine Learning
Source: Front Mol Biosci. 2021 Sep 22;8:677547. doi: 10.3389/fmolb.2021.677547 (PMC8493061; doi:10.3389/fmolb.2021.677547)

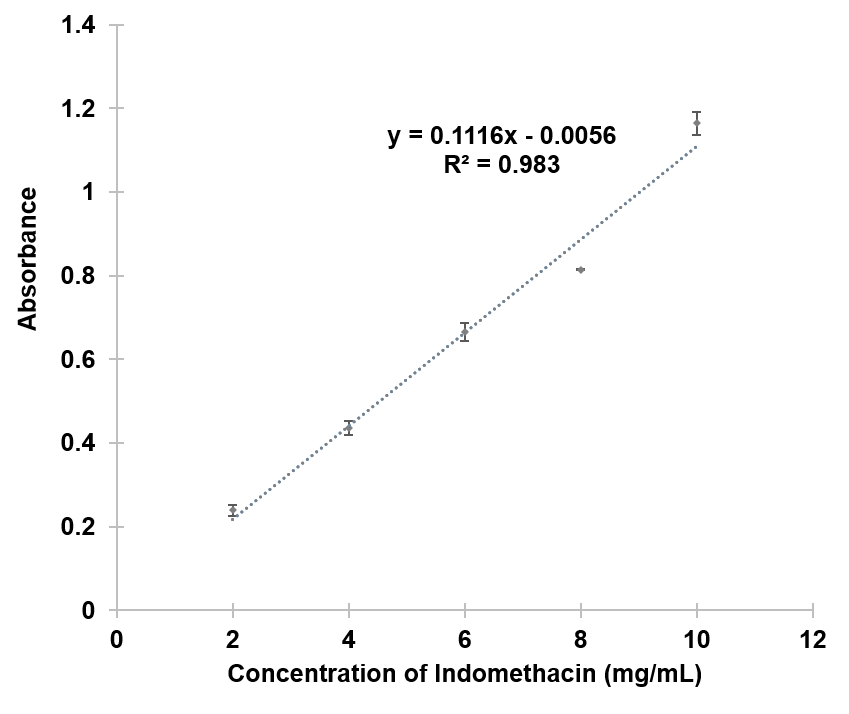

Supplement: Supplementary file 1 [file Image1.PNG]
